# Deciphering the Biological Activities of *Dunaliella* sp. Aqueous Extract from Stressed Conditions on Breast Cancer: from in Vitro to in Vivo Investigations

**DOI:** 10.3390/ijms21051719

**Published:** 2020-03-03

**Authors:** Fatma Elleuch, Patrick Baril, Mohamed Barkallah, Federico Perche, Slim Abdelkafi, Imen Fendri, Chantal Pichon

**Affiliations:** 1 Laboratoire de Biotechnologie Végétale Appliquée à l’Amélioration des Cultures, Faculty of Sciences of Sfax, University of Sfax, Sfax 3000, Tunisia; fatma.elleuch@ymail.com; 2 Centre de Biophysique Moléculaire (CBM), CNRS UPR 4301, 45071 Orleans, France; patrick.baril@cnrs.fr (P.B.); federico.perche@cnrs-orleans.fr (F.P.); 3 Unité de Biotechnologie des Algues, Ecole Nationale d’Ingénieurs de Sfax, University of Sfax, Sfax 3038, Tunisia; mohamedbarkallah@gmail.com (M.B.); slim.abdelkafi@enis.tn (S.A.)

**Keywords:** cancer, *Dunaliella*, microalgae, 4T1 cells

## Abstract

In order to harness local resources to improve well-being and human health, we aim in this study to investigate if the microalgae *Dunaliella* sp. isolated from the Tunisian coastal zone possesses any anticancer activity. *Dunaliella* sp. was cultured under normal (DSC) or stressed (DSS) conditions and extracted using different procedures. The biological activity assessment was performed on the Triple Negative Breast Cancer (TNBC) using 4T1 murine cells as a model. Results indicate that: (i) aqueous extract was the most cytotoxic compared to ethanolic and hydroalcoholic extracts; (ii) DSS activity was superior to that of DSC. DSS extracts induced apoptosis rather than necrosis, as evidenced by DNA fragmentation, PARP-1 cleavage and caspase-3 activation. Evaluation in an orthotopic TNBC model validated the anticancer activity in vivo. Intratumoral injection of DSS extract resulted in reduced tumor growth and an enhanced immune system activation. On the transcriptional side, the expression level of the immunosuppressive enzyme *Arg-1* was decreased, as well as those of *NOS-2* and *COX-2* genes. These results suggest a potential anticancer activity of Tunisian *Dunaliella* sp. deserving further attention.

## 1. Introduction

Microalgae represent an important source of biologically active compounds. *Dunaliella salina* is an interesting microalga as it is characterised by its ability to produce diverse metabolites depending on the culture conditions. This green microalgae is well known for its high production of carotenoids, especially β-carotene, with production reaching up to 10–14% dry weight, but also polyunsaturated fatty acids, glycerol and other antioxidants [1]. Thanks to these features, *Dunaliella* is on the list of products authorized for food supplementation and their conditions of use according to French law (NOR: ERNC1406332A, 24 June 2014). *Dunaliella* is a source of the food supplement *E160* (β-carotene). There are some studies reporting the effect of *Dunaliella* in whole powder form against fibrosarcoma [2], colitis [3], DMBA-induced breast cancer [4], thanks to its immunological and anti-leukemic properties [5]. Further studies have focused on identifying the active compounds in *Dunaliella* extracts. Other studies have been performed with fractions extracted with different solvents like hexane, ethanol and methanol, mainly used in order to extract β-carotene or to purify it. Those extracts have been studied to assess their antioxidant activity [6,7], antimicrobial activity against pathogens isolated from patients with external otitis [8], inflammatory and immune response [9,10,11]. Concerning anti-cancer activity, their effect in lung carcinoma [12], leukemic cancer [13], cutaneous carcinoma [14], prostate cancer [15], breast carcinoma [16], and oral squamous carcinoma [17] have been assessed. The majority of these extracts were shown to induce cell cycle arrest at G0/G1 phase and apoptosis cell death.

Breast cancer is the most common form of cancer amongst women around the world contributing to 25.4% of the total number of new cases diagnosed in 2018 according to the World Cancer Research Fund and American Institute for Cancer Research [18]. Breast cancer is subdivided into four groups depending on the presence or the absence of three receptors for oestrogen, progesterone and human epidermal growth factor receptor 2 (HER-2) [19]. The type with the worst prognosis is Triple Negative Breast Cancer (TNBC), characterized by the total absence of these three receptors, which is extremely difficult to handle by either hormonal and/or targeted therapies [20]. These aggressive tumors represent 12 to 17% of cases and are characterised by an elevated nuclear grade, mitotic activity and high power to metastasize to the viscera [21]. Many treatments are currently proposed comprising the use of PARP inhibitors to induce cancer cell death [22].

In healthy cases, mechanisms of cell death, such as apoptosis (programmed process), necrosis (accidental process) and autophagy are the gatekeepers responsible for maintaining homeostasis of the organism. Hallmarks of cancer include selective growth and proliferative advantages, altered stress responses favouring overall survival, vascularization, invasion and metastasis, metabolic rewiring, an abetting microenvironment, and immune activity [23].

Apoptosis is triggered through the intrinsic or mitochondrial pathway by activation of caspase-9 and in the extrinsic pathway by caspase-8 activation. The perforin/granzyme pathway also leads to caspase-3 activation [24]. Candé et al., have described an independent caspase pathway through the translocation of Apoptosis-Inducing Factor (AIF) to the nucleus causing chromatin condensation [25]. Effector caspases-3, 6 and 7 cleave various substrates such as PARP, cytokeratins, the nuclear protein NuMA, the plasma membrane cytoskeletal protein α-fodrin, etc., resulting in biochemical modifications such as protein cleavage and DNA breakdown; and morphological changes such as cell shrinkage, pyknosis, karyorrhexis and membrane blebbing [24]. Thereby, PARP-1 cleavage (deactivation) is considered as a marker of apoptosis [21]. In fact, PARP-1 is the most studied protein of PARP-family, containing at least 18 members, as it represents approximately 85% of the total activity of cellular PARP [26,27]. Its major role is to repair DNA damage by detecting single stranded DNA to maintain genomic integrity. PARP-1 is composed of a 54 KDa catalytic domain, a 46 kDa DNA Binding Domain (DBD) and a 22-kD auto-modification domain (AMD) [27]. It is a substrate of caspase-3 and 7, calpains, cathepsins, granzymes and matrix metalloproteases creating various exposed structural domains that subsequently induce specific forms of cell death [27]. As a substrate of caspase-3, 7, cathepsin B and cathepsin D, it can be cleaved into 89 and 24 Kda fragments.

Cancer cells have the ability to escape both apoptosis and immunological surveillance [28,29]. The goal of cancer immunotherapy is to reinforce the immune system and to restore such immunosurveillance. The presence of tumor-infiltrating lymphocytes (TILs) is positively correlated with prognosis [30,31,32,33], depending obviously on the identity of infiltrating immune cells. If these infiltrates are CD8^+^ T lymphocytes or NK cells, they will have a beneficial effect by enhancing tumor destruction [34,35]. CD4^+^ T-helper 1 cells (Th1) are also involved in the orchestration of antitumor responses by activating the antigen presenting cells as dendritic cells and enhancing CD8 T lymphocyte infiltration through IFN-γ, TNF-α and IL-2 production [36]. One of the features of tumor cells is their ability to produce a specific environment favouring the infiltration of immunosuppressive cells such as Treg (immunosuppressor), Th2 (T helper CD4^+^ type 2) and under some circumstance Th17 (proinflammatory), M2 macrophages, N2 neutrophils and myeloid-derived suppressor cells (MDSC) [34,36,37]. For breast cancer, there is a positive correlation between the presence of TILs and the improvement of clinical outcomes [30]. To our knowledge, this study reports for the first time how an aqueous extract of an isolated strain *Dunaliella* sp is effective against breast cancer by inducing cell death pathways. The study combines both in vitro and in vivo experiments performed with 4T1 aggressive TNBC.

## 2. Results

### 2.1. 4T1 Mammary Carcinoma Cells Sensitivity towards DS Extracts

**Figure 1:**
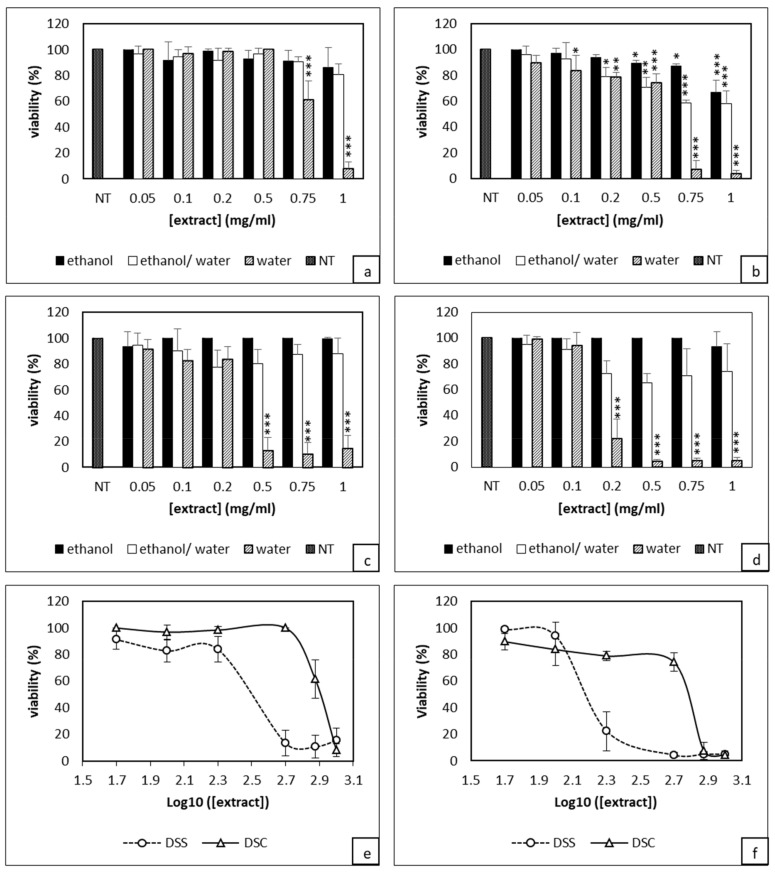
Evaluation of *Dunaliella* extracts from normal (DSC) or stressed (DSS) conditions effect on 4T1 cells viability through XTT assay. Cells were treated with either DSC (**a**,**b**) or DSS (**c**,**d**) for either 24h (**a**,**c**) or 48h (**b**,**d**). Estimation of IC_50_ values after 24h (**e**) and 48 h (**f**) of DSC and DSS treatments. Results shown are mean values ± standard deviation of three independent experiments (*** *p* <0.001; ** *p* < 0.01; * *p* < 0.05) according to One-way ANOVA using XLSTAT.

The first set of experiments was to determine the effect of DS extracted from different procedures on the viability of 4T1 cells. When cells were treated by DSC aqueous extract at 1 and 0.75 mg/mL during 24 h, the cell viability was significantly reduced to 10% and 60%, respectively (Figure 1a). Lower doses did not show any effect. After 48 h, the cytotoxicity was more pronounced, as only 4% of cells treated with 1 mg/mL of DSC survived with a significant reduction of cell viability for all doses except for a 0.05 mg/mL dose (Figure 1b).

In contrast, ethanolic and hydroalcoholic extracts showed no significant effect after 24 h treatment. The ethanolic extract used at 1 mg/mL only became cytotoxic after 48 h with 40% of dead cells compared to controls (Figure 1b). Similar data were obtained with both 1 and 0.75 mg/mL of DSC hydroalcoholic extract. However, for cells treated with 0.5 and 0.2 mg/mL, there was only 20% of cytotoxicity compared to control cells. Concerning DSS-based experiments, ethanolic and hydroalcoholic extracts did not show any antiproliferative activity after either 24 or 48 h treatment. By contrast, the aqueous extract exhibited a very high cytotoxicity. At 0.5 mg/mL, almost 90% cytotoxicity was observed after 24 h (Figure 1c). As for DSC, this effect was further enhanced after 48 h treatment; DSS at 0.2 mg/mL became cytotoxic with only 22% of viable cells; for higher concentrations, only 4% of live cells were left (Figure 1d). As observed for DSC, cell treatment with 0.1 and 0.05 mg/mL of DSS did not result in any significant effect on the cell viability. Overall, the above data indicate that aqueous extract was superior to the ethanolic and hydroalcoholic extracts. DSC and DSS aqueous extracts act in a dose-dependent-manner. After 24 h treatment, the IC_50_ values of DSC and DSS aqueous extracts were 0.804 ± 0.046 mg/mL and 0.317 ± 0.026 mg/mL, respectively (Figure 1e). After 48 h treatment, these concentrations decreased to 0.608 ± 0.008 mg/mL and 0.149 ± 0.008 mg/mL (Figure 1f). Next, we performed a Crystal Violet staining known to bind exclusively to proteins and DNA of viable cells remaining attached to the plate [38]. Crystal Violet staining nicely demonstrated the superiority of DSS over DSC extracts (Figure S1). Overall, this first set of data show that aqueous extract of DSS has a cytotoxic activity which is dose and time dependent.

### 2.2. Deciphering DSS Cytotoxicity Mechanism 

Next, we performed different experiments to assess through which mechanism DSS might act to induce the cytotoxicity towards 4T1 cells.

#### 2.2.1. Qualitative Evaluation by Tunel Assay 

**Figure 2:**
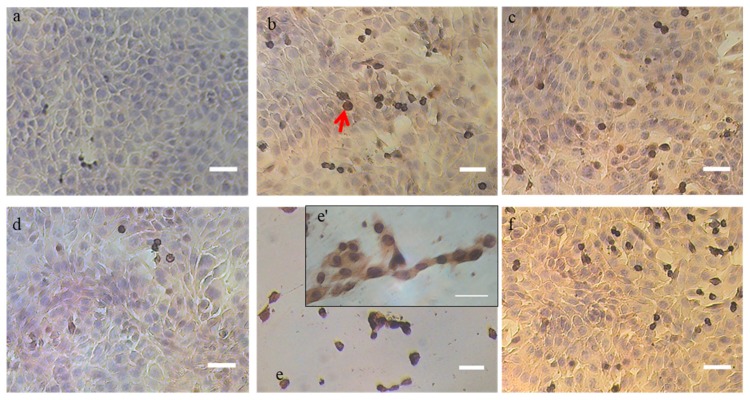
Detection of apoptotic cells in situ by DNA fragmentation through TUNEL assay. The apoptotic cells are stained dark brown (red arrows) after counterstaining with hematoxylin. (**a**) untreated cells at 10X magnification, (**b**) DNase-treated cells for 20 min at 10U (positive control) at 10X magnification, (**c**) 0.5 mg/mL DSS treated cells after 18 h at 10X magnification, (**d**) 0.5 mg/mL DSC-treated cells after 18 h at 10X magnification, (**e** and **e’**) 0.5 mg/mL DSS treated cells after 24 h observed at 10X and 40X magnification, respectively, (**f**) 0.5 mg/mL DSC-treated cells after 24 h at 10X magnification. Note: Scale bar = 50 µm

We first performed a TUNEL assay on DSS- and DSC-treated cells to qualitatively detect the presence of apoptotic cells. As shown in Figure 2, intense brown staining of cell nuclei was observed in 4T1 cells treated with 0.5 mg/mL DSS extract as soon as 18 h post-incubation. 

The apoptotic effect of the DSS extract on the 4T1 cells was even more pronounced 24 h post incubation and was similar to DNase-treated cells. Less nuclei were stained in cells treated with DSC extract compared to DSS treatment at these early and late time points albeit more than for non-treated cells.

#### 2.2.2. Qualitative evaluation by Western blot

**Figure 3:**
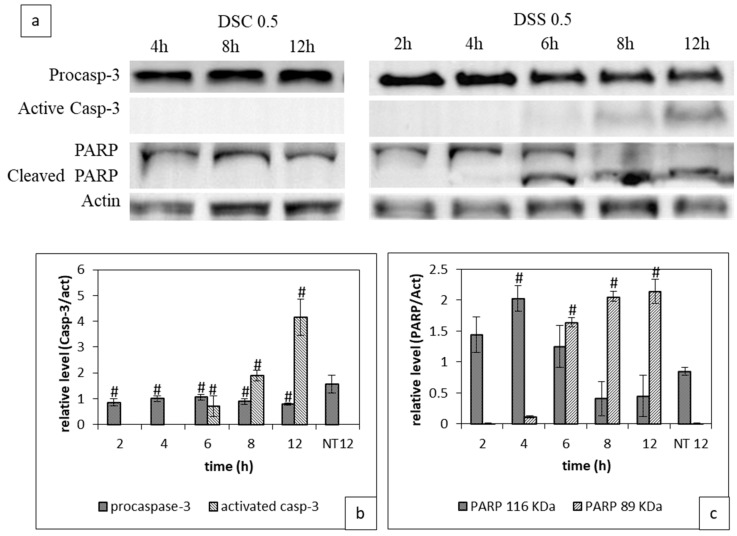
Impact of DSC and DSS treatment on Caspase-3 activation and PARP-1 cleavage. Cells were treated with either DSC or DSS at 0.5 mg/mL during 2 to 12 h before processing for western blot analysis as described in Material and Methods. (**a**) PARP-1 uncleaved and cleaved form, pro and active Caspase-3 immunoblotting (**b**) Full-length western blot images giving details are available in Figure S2; Quantitative evaluation of the expression of pro and caspase-3, (**c**) Quantitative evaluation of the expression of PARP-1 at 116 and 89 KDa. Results shown are mean values ± standard deviation of three independent assys through Image J (^#^ *p* < 0.05) according to one-way ANOVA using Origin 8.

We investigated the activation of caspase-3 proteins and the deactivation of PARP-1 involved in apoptosis mechanism in cells treated with 0.5 mg/mL DSC and DSC. Western blots, shown in Figure 3, reveal that caspase-3 was activated in cells treated with 0.5 mg/mL DSS extract. The active form of caspase-3 was detected from 6 h and it increased over time. Concomitantly, the cleaved form of PARP-1 was produced starting at 6 h and it rose over time. At 12 h post-treatment, the cleaved PARP-1 form was detected majorly. Cell treatment with 0.5 mg/mL of the DSC extract during 12 h did not lead to any induction of the apoptotic effects of these extracts. Taken together, this blots data confirms again the superiority of DSS over DSC. 

### 2.3. Tumor Growth Inhibition by Dunaliella Extracts 

**Figure 4:**
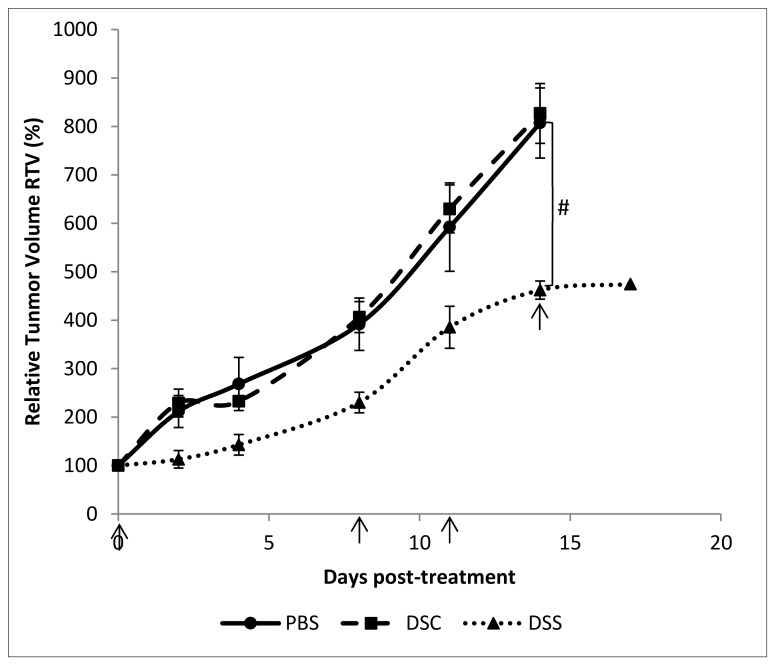
Relative tumor volume of mice bearing 4T1 cells orthotopically implanted and treated with DSC and DSS aqueous extracts at 5 mg/kg (10, 18, 21 and 24 days post-implantation). Results shown are mean values ± standard error of the mean (^#^ *p* < 0.05) according to one-way ANOVA using Origin 8.

Last, we sought to confirm the in vitro results by investigating whether aqueous *Dunaliella* extract could effectively suppress growth tumor in vivo. 4T1 breast mammary cells were orthotopically implanted into the mammary glands of Balb/c mice. Ten days post-implantation, mice were treated by intra-tumor injection of either DSC or DSS aqueous extract at the dosage of 5 mg/kg. As illustrated in Figure 4, mice treated with DSC extract and PBS have similar tumor growth trends. By contrast, DSS aqueous extract treatment significantly reduced the tumor size compared to control mice (*p* < 0.5). The relative tumor volume (RTV) was two times lower than those of control mice. There was also a delay in the development of the tumor over time, which allowed us to extend the treatment by 3 days before animal sacrifice in this group. It worth noting that none of the mice did lost weight during this follow-up period (Figure S3).

### 2.4. Assessment of Immune Activation 

**Figure 5:**
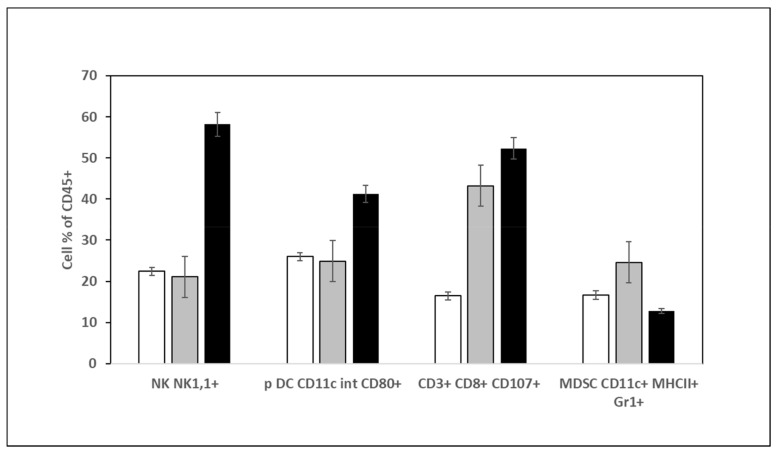
Quantification of immune cells in the tumor of mice treated either with PBS, DSC or DSS at 5 mg/kg. Cells extracted from tumors were processed for immunophenotyping and analyzed by flow cytometry. White: Non-treated mice; grey: DSS treated mice; black: DSC treated mice.

This last decade, it has been demonstrated that cancer development is associated with immune system modulation *via* the tumor immunosuppressive microenvironment [39]. We wanted first to check whether aqueous DSS and DSC extract treatments could trigger an immunostimulatory activity by evaluating the types of tumor-infiltrating immune cells. Figure 5 shows that compared to tumors of mice treated with PBS or DSC extract, those of mice treated with DSS aqueous extract contained a higher infiltration of NK (NK1,1^+^) cells, more activated T cells (CD3^+^, CD8^+^, CD107^+^) and plasmacytoid dendritic cells (CD11c^+^int CD80^+^) against a decrease in myeloid derived suppressor cells (MDSC: CD11c^+^MHCII^+^Gr1^+^).

**Figure 6:**
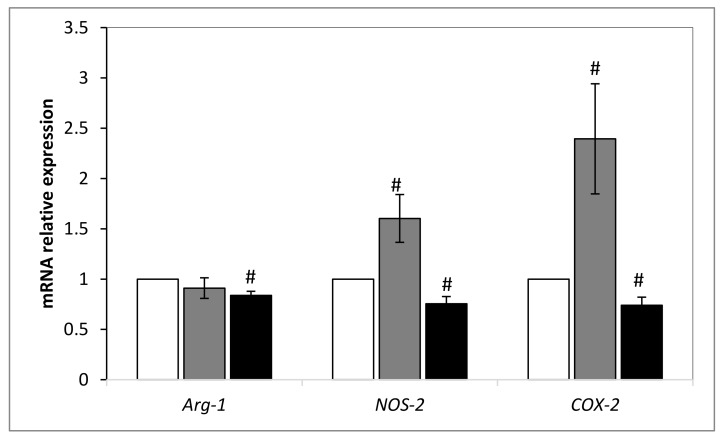
Relative expression level of *Arg-1*, *NOS-2* and *COX-2* after DSC and DSS treatment by normalization against *GAPDH* gene. Results shown are mean values ± standard error of the mean where (^#^ *p* < 0.05) indicates a significant difference between gene expressions among mice treated with PBS and those extract-treated, according to the one-way ANOVA test followed by Tukey test using Origin 8. White: Non-treated mice; grey: DSS treated mice; black: DSC treated mice.

Second, we investigated how could be modulated the expression of *arginase-1 (Arg-1)*, *cyclooxygenase 2 (COX-2)* and *nitric oxide synthase-2 (NOS-2)* genes in DSS-treated tumors compared to non-treated ones. As shown in Figure 6, there was a transcriptional modulation of *Arg-1*, *NOS-2* and *COX-2* expression in treated tumors. A small but significant decrease of *Arg-1* expression was found in DSS-treated tumors compared to DSC and PBS-treated ones. The expression of *NOS-2* and *COX-2* couple was diminished significantly after DSS treatment. By contrast, their expression was significantly increased in DSC-treated tumors with no modulation of *Arg-1*. Those data are in line with those found in Figure 4 and confirm that DSS extract holds a potential anti-tumor activity by acting on immune cells and on the metabolism. 

## 3. Discussion

This study was performed with an isolated *Dunaliella* sp. strain from the Tunisian coast identified to have 97% of similarity with *Dunaliella salina* [40]. *Dunaliella salina* is known for its high production of antioxidants, mainly β-carotene, which leads to its anticancer activity [4,5,12,13,14,15,16,17]. *D. salina* is also able to produce hydrophilic compounds such as flavonoids, tannins and alkaloids [41]. We looked for the possible anti-cancer activity of our strain cultivated under standard or non-stressed condition (DSC) or a stressed condition (DSS) in order to enhance carotenoids and antioxidant productivity as reported previously [14]. Flavonoids and phenolic compound levels can be also increased in stressed conditions of nitrogen deficiency and UV B irradiance, in addition to the production of antioxidant enzymes and glutathione [42]. DSC and DSS were extracted by maceration either in ethanol, ethanol/water (V/V) or in distilled water followed by a sonication step. Extraction in water led to the most active extracts against murine breast carcinoma 4T1 cells suggesting that compounds responsible for this cytotoxicity were hydrophilic. The fact that DSS extract was almost four times more toxic than DSC one could be explained either by an over accumulation of those compounds under stressed conditions and a better lysis of stressed over non-stressed cells by the sonication step. Indeed, *Dunaliella* lacks a rigid cell wall and can be lysed by osmotic shock [43]. It is interesting that aqueous extraction bore higher biological anti-cancer activity compared to other solvents. Water has been widely used as a solvent for plants and other green microalgae extractions, thanks to its “safe” nature. The extract obtained is easy for freeze-drying and thereafter soluble in aqueous solution as culture medium. Thus, it avoids any residual cytotoxic effect of solvents and the need of solubilising the extract in DMSO as for ethanolic extract. 

An aqueous extract of *Chlorella vulgaris,* which is a green microalga has been reported to have a significant anti-proliferation activity towards Ehrlich ascites carcinoma (EACC) cells and human hepatocarcinoma HepG2 cells, respectively [44]. Sonicated aqueous extract of a mixture of microalgae (not identified specifically) have been shown to be effective against the proliferation of different types of cancer cells from lung (A549, H460), prostate (PC3, DU145), stomach (N87), breast (MCF7), pancreas (BxPC3) and bone (MNNG) [45]. The present study reports for the first time that *Dunaliella* aqueous holds an efficient antitumor activity against breast cancer cells growth both in vitro and in vivo by intratumoral injection. Previous reports related on the use of *D. salina* were performed with ethanolic extract or the whole powder with an oral administration requiring at least more than two weeks of treatments with high doses from 300 mg/kg up to 1000 mg/kg [2,4,5].

The ethanolic and hydroalcoholic extracts of DSC were found to be active, which is in correlation with previous cellular studies performed in A549 lung cancer cells [12], A431cutaneous carcinoma cells [14], HL-60 and MV-4-11 leukemia cells [13]. In contrast, DSS ethanolic extract was not efficient, suggesting that the procedure led to the extraction of some compounds that may stimulate cell growth. DSS ethanolic extract likely contained high amount of β-carotene, tocopherol and ascorbic acid compared to DSC as reported by [14]. Paolini et al., have shown that β-carotene is rather associated with a significant increase in the incidence of lung cancer, cardiovascular disease and mortality in smokers and asbestos workers [46,47]. This property depends also from the ratio of the 9-*cis*/all-*trans* β-carotene form and greater this ratio is, the better the antioxidant and antiproliferative power of β-carotene will be [48]. 

In this study, we decided to go deeper in the investigation of the cell death mechanism induced by DSS extract. TUNEL assay labelling the 3′OH ends of apoptotic nucleus showed an intense brown staining of nucleus of 4T1 cells treated with DSS extract, which means that the extract triggers cell death by apoptosis. This result was confirmed by caspase-3 activation and PARP-1cleavage [49]. Caspase-3 activation and PARP-1 deactivation were found in cells treated with DSS extract in 12 h treatment with DSS but not with DSC. This difference is in accordance with the cytotoxicity assay. Activated caspase-3 cleaves PARP-1 into two fragments of 89 and 24 KDa. The fragment of 24 kDa binds to the damaged DNA irreversibly through two zinc finger motifs and acts as a *trans*-dominant inhibitor of active PARP-1 and other DNA repairing enzymes [27]. When cells have a severe damage of DNA, PARP-1 activity must be amplified which results in an increase of the reparative form of PARP-1 (un-cleaved formed) as we observed after 4 h treatment with DSS. If this activity is not controlled, it leads to a passive necrotic cell death resulting from prolonged ATP depletion [27]. In this study, the reparative process was blocked by caspase activity that cleaves PARP-1 as observed after 6 h of treatment. Cells were then induced to apoptotic death. It is worth to note that for TNBC, DNA damage of cancer cells was observed during chemotherapy and inhibiting PARP-1 is important to block DNA repair mechanisms [21]. 

Data obtained from in vivo experiments confirmed the growth-inhibitory effect of DSS aqueous extract on murine breast carcinoma cells. We decided to treat the mice by intra-tumoral injection instead of oral administration that requires a high dosage extract with daily intake, rather difficult to master, as reported in previous studies [2,3,4,5]. The treatment with 5 mg/kg of DSS extract led to a delay of tumor growth rate and a significant reduction of tumor volume without affecting the weight of mice. Knowing the impact of the immune system in cancer growth and the possible adjuvant effect of phytochemicals, we conducted an analysis of immune cells amount inside tumors. It was proven previously that treatment of rat hepatocellular carcinoma with β-carotene inhibited significantly tumor growth, enhanced NK cells infiltration as well as production of IL-2 and TNF-α, resulting in an improved clinical outcomes as attested by significant decrease of blood alanine aminotransferase, aspartate aminotransferase and alkaline phosphatase activities [50]. Moreover, carotenoids influence immune function by regulating membrane fluidity and gap-junctional communication [51]. Finally, flavonoids also have anti-proliferative effects on cancer cells through suppression of the PI3k/Akt/mTOR pathway and activation of T CD4+ [52]. In our study, we show for the first time that DSS extract treatment could result in an increase (3-fold) of NK recruitment inside the tumors compared to non-treated and DSC treated tumors. The major recruited cells were NK cells, which are part of the innate immunity and having a crucial role in immunosurveillance mechanism. Those cells induce a cell death by triggering apoptosis independent or dependent caspase using two mechanisms that are the exocytosis of cytotoxic granules containing perforin and granzyme or the induction of apoptosis *via* death receptors [50]. NK can be activated by natural compounds such as vitamins A, B, C, D, and E, lectins, polysaccharides, and other phytochemicals [50], therefore, microalgae extracts contain various compounds that could likely activate NK cells. A similar effect has been reported previously with WEHI-3-induced leukemic mice treated with oral administration of 922 mg/kg of *D. salina* during 2 weeks [5]. We observed that pDC number was almost doubled in DSS-treated tumors compared to non-treated and DSC-treated tumors. Those cells could play a role in immunosurveillance of tumors. Indeed, the activation of those cells could induce a large production amount of type I interferon (IFN) [51]. In fact, IFNs can induce apoptosis of cancer cells by direct cytotoxic effects or *via* enhancement of death-inducing molecules [52]. Our data highlight as well that the number of CD8^+^ T cytotoxic T lymphocytes, another type of key cells in the antitumor response, was also enhanced (~3-fold). Those cells belong to the adaptive immunity system and are activated by NK cells [28]. The rise in CD8^+^ T cells infiltration was seen to be associated with a good prognostic among breast cancer patients [53]. Recently, an interesting study reported that activated pDCs were able to kill 4T1 breast cancer cells through TRAIL and granzyme B death-initiating molecules [54]. Authors have established that pDC were the initiator of the sequential activation of NK and CD8^+^ T cells; both increased upon DSS treatment. Our results shows also a decrease in number of myeloid-derived suppressor cells (MDSC) in the tumor, which are immature myeloid cells with potent immunosuppressive potential [55].

Since metabolic changes occur during cancer development, we checked the modulation of *Arg-1*, *NOS-2* and *COX-2* expression, three key enzymes involved in tumorigenesis. Our data reveal a reduced expression of *Arg-1* and *NOS-2* in tumors treated with DSS extract. This immunosuppressive enzyme is produced both by cancer cells and polymorphonuclear neutrophils and M2 macrophage subtype. It transforms l-arginine to l-ornithine and urea [56]. The depletion of l-arginine from the tumor microenvironment inhibits the re-expression of the CD3 ζ chain [57] and induces the down- regulation of T cell receptor making T cells anergic [55]. l-Arginine is also catabolized by iNOS (or NOS-2) that produces nitric oxide (NO) leading as well to an induction of T cell anergy [55]. Therefore, the transcriptional level decrease of these two genes influences positively the function of T cells. The inhibition of iNOS has been reported to significantly reduce tumor growth, lung metastases and tumor initiation [58]. Another predictive biomarker of immune inhibition is COX-2. It is involved in the transformation of arachidonic acid to prostaglandin. Its activity can suppress DC, NK, T cells, type-1 immunity and promotes type-2 immunity [59]. Therefore, the decrease of its expression in DSS-treated tumors is coherent with the enhancement of anti-tumor immunity. Note that the enzymatic activity of COX-2 has been found to be reduced in DMBA induced mammary cancer cells treated by *D.salina* powder [4]. Nassar et al. have shown that high expression of COX-2 in breast carcinoma was positively correlated with tumor size and tumor grade [60]. It is important to point out that the crosstalk between NOS-2 and COX-2 occurred *via* their respective key products NO and PGE-2. Simultaneous inhibition of those two genes by aminoguanidine and aspirin/ indomethacin resulted in a significant reduction of the growth of a xenograft murine breast cancer model [61]. 

The findings of this study have to be seen in light of some limitations. The activity of the extract on normal cells would be interesting to assess, but it deserves more complete work that is beyond the topic of our current study. Moreover, as we intentionally choose the intratumoral route of administration to concentrate the extracts in the targeted tissues; it is unlikely that the extract would diffuse outside of the tumour site. This administration route allows a maximal concentration of the extract in the targeted tumour tissues and, consequently, a maximal anti-tumoral effect. Such local intra-tumor injection is widely used for targeted therapy as immunotherapy [62,63]. Despite these advantages, there is some restriction because only accessible sites of sufficient size can be injected [64].

## 4. Materials and Methods 

### 4.1. Cancer Cell Line Culture 

4T1 mouse mammary tumor cells (ATCC^®^ CRL-2539™, Rockville, MD, USA), were grown as monolayers, at 37 °C in 5% CO_2_–95% air humidified atmosphere. They were cultured in Roswell Park Memorial Institute (RPMI) 1640 medium supplemented with 10% fetal bovine serum (*v*/*v*), penicillin 100 U.mL^−1^ and streptomycin 100 μg.mL^−1^. The medium was purchased from Sigma-Aldrich (St. Quentin Fallavier, France). 

### 4.2. Microalgae Culture 

*Dunaliella* sp. having 97% of similarity with *D. salina, D*. *quartolecta* and *D*. *polymorpha* was isolated from the Sebkha of Sidi El Hani (Sousse, Tunisia) [40]. It was cultivated in F/2 medium based on artificial sea water (ASW). Cells were grown under unstressed (DSC) and stressed (DSS) conditions as previously optimized by Elleuch et al. [65].

### 4.3. Extracts Preparation 

In order to extract various molecules from the lyophilized powder of the isolated strain, three extracts of different polarities were prepared according to a modified protocol of [66] with either in ethanol, water and ethanol/water (1/1, *V*/*V*). For ethanol-based extraction, 1 g of DSC or DSS freeze-dried microalgae, were added to 100 mL ethanol, ground until well homogenized and incubated for 2 h at 4 °C in darkness. For water extract, a sonication step for 15 min at 40 kHz was added, and then the mixture was centrifuged at 5000× *g* for 10 min at 4 °C. The extraction was repeated twice to recover the maximum amount of compound. Ethanol extract was concentrated by evaporation under a nitrogen flow to dryness in the dark while the water and ethanol/water extracts were freeze-drying. All dry extracts were stored at –20 °C until use.

### 4.4. Cytotoxicity Evaluation

A XTT kit (Cell Proliferation Kit II, Roche) from Sigma-Aldrich (St. Quentin Fallavier, France) assay was performed to determine the cytotoxicity of the different extract prepared. This assay is based on the property of living cells to reduction of the yellow 2,3-bis-(2-methoxy-4-nitro-5-sulfophenyl)- 2*H*-tetrazolium-5-carboxanilide tetrazolium salt (XTT) into a soluble derivative of brightly orange formazan by mitochondrial oxidoreductases enzymes, so the coloration intensity is directly correlated to the number of living cells. 

In brief, approximately 1 × 10^4^ cells/well were seeded onto 96 wells plate. After 24 h, the medium was discarded and cells were treated for 24 and 48 h with fresh medium containing different concentrations (from 0.05 mg to 1 mg/mL) of indicated extracts from DSC and DSS. Ethanolic and 50% hydroalcoholic dried extracts were previously dissolved in culture medium containing 2% DMSO. Note that since ethanolic and hydroalcoholic extracts were dissolved in 2% DMSO, control cells were treated with the medium containing the corresponding concentration of DMSO without the extract. For aqueous extracts were diluted directly in the medium, controls cells were treated with medium without the extract.

After incubation, the medium was discarded, cells were washed twice with PBS and 100 µL of XTT mixture was added and incubated for 3h. The absorbance was measured for each well at wavelength 450 nm and 650 nm using a multilabel microplate reader Victor spectrophotometer (PerkinElmer, Waltham, MA, USA).

The percentage of viability was calculated according to the manufacturer’s instructions. As negative controls for aqueous and ethanolic/hydroethanolic extracts conditions, cells were incubated in culture medium (RPMI) containing 2% DMSO. The absorbance of the medium alone is also considered as the blank for the test. Each condition was tested in triplicate. 

### 4.5. Cristal Violet Assay

4T1 cells were seeded at 1 × 10^5^ cells/well in 24 well plates one day before the experiments. Cells were then incubated in presence of different extracts at indicated concentration. After 48 h of treatment, cells were washed twice with PBS and fixed with cold methanol for 10 min. Fixed cells were washed and stained with Crystal Violet (0.5% w/v prepared in 25% methanol) for 10 min at room temperature. The wells were washed until complete discoloration of the supernatant.

### 4.6. Tunel Assay 

TdT-mediated dUTP nick end labeling (TUNEL) is an adequate assay for the detection of apoptotic cells as it labels the 3’ OH ends of fragmented DNA in situ on dark brown nuclei by peroxidase staining. The TUNEL assay was conducted using the DeadEnd Colorimetric TUNEL System (Promega, Madison, WI, USA) as described in the manufacturer’s instructions with minor modifications of directly staining cells in 48 well plates. Stained sections were analyzed with a light microscope (Nikon Diaphot 300).

Positive control cells were generated by treating fixed cells with DNase I (Sigma) at 10U for 20 min, whereas negative controls are those without terminal TdT enzyme in the TUNEL reaction mixture.

Briefly, 5 × 10^4^ cells/well were seeded onto 48 well plates and cultured at 37 °C for 24 h. After treatment with DSS and DSC at 0.5 and 0.2 mg/mL for 24 h, cells were washed twice with PBS and fixed with 4% paraformaldehyde for 20 min at room temperature (RT). Cells were permeabilized with 0.02% Triton X-100 for 5 min at RT. After calibration with equilibration buffer, cells were treated with rTdT Reaction Mix for 1h at 37 °C in a humidified chamber. SSC buffer 1X was applied for 15 min before quenching endogenous peroxidase with 0.3% H_2_O_2_. Cells were incubated with streptavidin HRP for 30 min at RT. The color development was done with DAB substrate, DAB chromogen and hydrogen peroxide. Finally, cells were washed with deionized water and counterstained with hematoxylin before microscopy analysis (Nikon Diaphot 300).

### 4.7. Western Blot Experiments

To investigate the cell death pathway, total proteins were extracted after cells treatment for 2, 4, 6, 8 and 12 h. Cells were washed twice with ice-cold PBS and lysed using radioimmune precipitation assay (RIPA) buffer containing 1% of protease inhibitor cocktail and 1% of phenylmethylsulfonyl fluoride (PMSF) by vortexing the lysate for 3 min. Proteins were recovered in the pellet after centrifugation at 12500 rpm for 10 min at 4 °C. Their concentration was determined using BCA assay (Protein Assay Kit (Interchim, Montlucon, France) and 20 μg of total proteins were resolved by gel electrophoresis under denaturing conditions SDS-PAGE. Depending on the molecular weight of the proteins to be detected, two types of gel were prepared: the first one at 10% for PARP-1 (116 and 89 KDa) and caspase-9 (37 KDa) and the second one at 14% for caspase-3 (17 KDa). The proteins were heated for 10 min at 95 °C in the presence of Laemmli buffer as a denaturant before loading in the wells of the gels. The migration was performed for 3 h at 150 V and 100 V for the first gel and the second, respectively. Upon migration, the proteins are electrotransferred on a polyvinylidene fluoride (PVDF) membrane by Trans-Blot^®^ Turbo ™ Transfer System (Bio-Rad, Hercules, CA, USA) (15 min, 25 V). The membranes were first treated with 5% non-fat dry milk in TBST (Tris-buffered saline, 0.05% Tween 20) for 1 h at RT. Then, they were incubated overnight with the indicated primary antibodies at 4 °C: β-actin (ab1801; 1:5000), PARP-1 (# 9542), caspase-3 (# 6992) caspase-9 (# 9504). The last three antibodies were used at a concentration of 1: 1000 as recommended by Cell Signaling Technology (Danvers, MA, USA. http://www.cellsignal.com). For the detection of these primary antibodies, the membrane was incubated for 1 h at room temperature with the anti-rabbit secondary antibody conjugated to horseradish peroxidase (Enzo Life Sciences, Inc). After each incubation, membranes were washed three times for 10 min with TBST. The protein bands were visualized using ECL chemiluminescence (Bio-Rad) and revealed to the Syngene Pxi (Ozyme, Saint Cyr l’Ecole, France) gel imager controlled by the GeneSys acquisition software. The protein bands were quantified using image software j. bands intensity was normalized to β-actin.

### 4.8. In vivo Experiments

For animal experiments, mice housing was performed in our animal facility (accreditation number D-45-234-12, Chantal Pichon) according to the guidelines of the French Ministry of Agriculture for experiments with laboratory animals (Law 87848). A local ethical committee (Comité d’Ethique pour l’Expérimentation Animale, Campus d’Orléans, France, French Registration CECCO 03) approved experimental procedures; which did the French Ministry of Agriculture validate according to the document file APAFiS #1964.

A total of 15 females BALB/c mice of 8 weeks of age and about 20 g in weight, were purchased from Elevage Janvier (Le Genest St Isle, France). The mice were housed in the CBM animal facility, at 22 °C, a dual phase (12/12 h) in a humidity and temperature-controlled room under standard laboratory conditions. Mice were divided into 5 groups of 5 mice per cage and were acclimated and quarantined for a week prior to experimentation. This latter was carried out under the accreditation of the CBM laboratory (APAFiS #1964, C. Pichon). The mice were depilated 2 days before tumor implantation. After anaesthesia with isoflurane, the mice received 5 × 10^5^ of 4T1 cells in a total volume of 50 μL by subcutaneous injection into the mammary gland. 10 days after implantation and the development of a primary tumor (palpable aspect), we started the treatment by intra-tumor injection of DSC and DSS aqueous extracts at 5 mg/kg of mouse. As the extracts were diluted in PBS, the negative control group received 50 μL of PBS per injection. Subsequently, treatments were performed on days 10, 18, 21 and 24 post-implantation. Tumor growth was monitored by measuring tumor volume using a calliper. Tumor volumes were normalized respectively to the volume of the tumor at day 0 of treatment through Equation (1) expressing the relative tumor volume (RTV) [67]. Mice were also daily observed individually and weighed during the experiment. The animals were sacrificed by cervical decapitation at the end of the protocol:

(1) $$RTV=\frac{Tumor\;Volume\;at\;day\;X}{Tumor\;Volume\;at\;day\;0}\;\times \;100$$

### 4.9. Immune Cell Identification

Implanted tumors were harvested after 15 days of treatment for analysis of immune cells. Tumors were suspended in PBS and cut into small pieces of 3–4 mm then centrifuged at 350xg for 10 min. The pellets were subsequently suspended in collagenase I (Gibco: Thermofisher Scientific, Les Ulis, France) at a concentration of 1 mg/mL in presence of 3 mM CaCl_2_ and incubated for 1 h at 37 °C with agitation. The obtained suspension was filtered, washed with PBS and cells were suspended in fresh PBS. Cells were incubated for 1 h at RT in the dark with labelled antibody listed in Table 1 which are used at a dilution of 1/100 in 1% BSA. Before cell flow cytometry analysis (Fortessa x 20, Becton Dickinson Biosciences, Rungis, France), cells were filtered and suspended in PBS. Multicolor CompBeads (BD Biosciences, Rungis France) were used for the cytometer settings according to the manufacturer’s instructions.

### 4.10. RNA Extraction and qPCR Analysis 

Total RNA was extracted from harvested tumors by TRIzol® reagent (Invitrogen, Carlsbad, CA, USA) after cutting tissue into small pieces. For better grinding of tissues, they were incubated for 10 min at -80 °C before being ground with Precelyss 24 (Precelyss, Bertin, France) using the following program: 5500 vibrations, 3 cycles of 30 s each. One µg of the obtained RNA_t_ was used for the synthesis of cDNA using the kit RevertAid First Strand cDNA Synthesis (Thermo Scientific, MA, USA). qPCR was performed using TB Green™ Premix Ex Taq ™ (Tli RNaseH Plus) (TaKaRa, Shiga, Japan) according to the manufacturer’s protocol. Primers used are cited in the Table 2. For all experiments, we checked efficiency and specificity of primers. PCR reactions were run on StepOnePlus™ Real-Time PCR System. Fold changes were calculated through the equation given by [68] by normalization against *GAPDH* reference gene.

## 5. Conclusions

Data from this study confirm other studies reporting the anti-proliferative activity of *D. salina* extract. This report shows for the first time the potentiality that an aqueous extract of *D. salina* grown under stress conditions holds when delivered inside tumors at doses as low as 5 mg/kg. The immunomodulatory effect of *D. salina* is of importance and opens an avenue of the use of microalgae extract as an immune adjuvant for anti-breast cancer therapeutic strategies. 

## Figures and Tables

**Table 1:** List of antibodies used for immune cells identification.

Recognized Antigen	Fluorochrome	Clone	Reference BD Pharmingen
CD3	PerCP/Cy5.5	145-2C11	551163
CD8	BV510	341	742916
NK1.1	BV60	PK136	563220
CD11c	BV711	HL3	563048
MHCII	PE/Cy7	L243	335830
CD80	FITC	16-10A1	553768
CD107a	BV78	61D4B	564349
Gr-1	APC-Cy™7	RB6-8C5	557661

**Table 2:** Primers used for qPCR.

Genes (Mouse)	Primer Sequence (5′→3′)	Amplicon Size	References
*GAPDH*	TCTCCCTCACAATTTCCATCCCAG	--	[69]
	GGGTGCAGCGAACTTTATTGATGG		
*Arg-1*	CTCCAAGCCAAAGTCCTTAGAG	185	[70]
	AGGAGCTGTCATTAGGGACATC		
*NOS-2*	CCAAGCCCTCACCTACTTCC	127	
	CTCTGAGGGCTGACACAAGG		
*COX-2*	TGAGTACCGCAAACGCTTCT	169	
	CTCCCCAAAGATAGCATCTGG		

## Supplementary Materials

Supplementary materials can be found at https://www.mdpi.com/1422-0067/21/5/1719/s1.

## Abbreviations

DSS	*Dunaliella* sp. cultured under stressed conditions
DSC	*Dunaliella* sp. cultured under unstressed (control) conditions
TNBC	Triple negative breast cancer
PARP-1	Poly(ADP-ribose) polymerase-1
*Arg-1*	*Arginase-1*
*NOS-2*	*Nitric oxide synthase 2*
*COX-2*	*CycloOXygenase-2*
GAPDH	GlycerAldehyde 3-Phosphate DeHydrogenase
DMBA	7,12-Dimethylbenz(a)anthracene
HER-2	Human epidermal growth factor receptor 2
DBD	DNA binding domain
AMD	Auto-modification domain
MDSC	Myeloid-derived suppressor cells
TIL	Tumor-infiltrating lymphocyte
RTV	Relative tumor volume
DC	Dendritic cells
MHC	Major histocompatibility complex
PGE-2	ProstaGlandin E2
NO	Nitric oxide
ASW	Artificial sea water
DMSO	Dimethyl sulfoxide
PVDF	PolyVinyliDene Fluoride
RIPA	RadioImmune Precipitation Assay
PMSF	PhenylMethylSulfonyl Fluoride
BCA	BiCinchoninic Acid
SDS-PAGE	Sodium dodecyl sulfate-polyacrylamide gel electrophoresis
TBST	Tris-buffered saline Tween
RT	Room temperature
PBS	Phosphate buffered saline
NuMA	Nuclear mitotic apparatus protein-1
AIF	Apoptosis-inducing factor
TNF	Tumor necrosis factor
RPMI	Roswell Park Memorial Institute
