# Supplementary material for: Deciphering the Biological Activities of Dunaliella sp. Aqueous Extract from Stressed Conditions on Breast Cancer: from in Vitro to in Vivo Investigations
Source: Int J Mol Sci. 2020 Mar 3;21(5):1719. doi: 10.3390/ijms21051719 (PMC7084689; doi:10.3390/ijms21051719)
Supplement: Supplementary file 1 [file ijms-21-01719-s001.pdf]

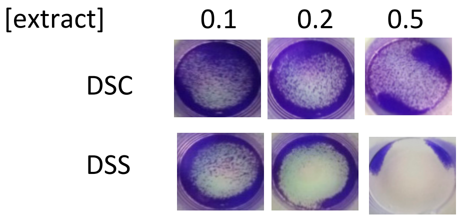

Figure S1. Representative images of Crystal violet staining of cells treated with either DSC or DSS aqueous extracts.

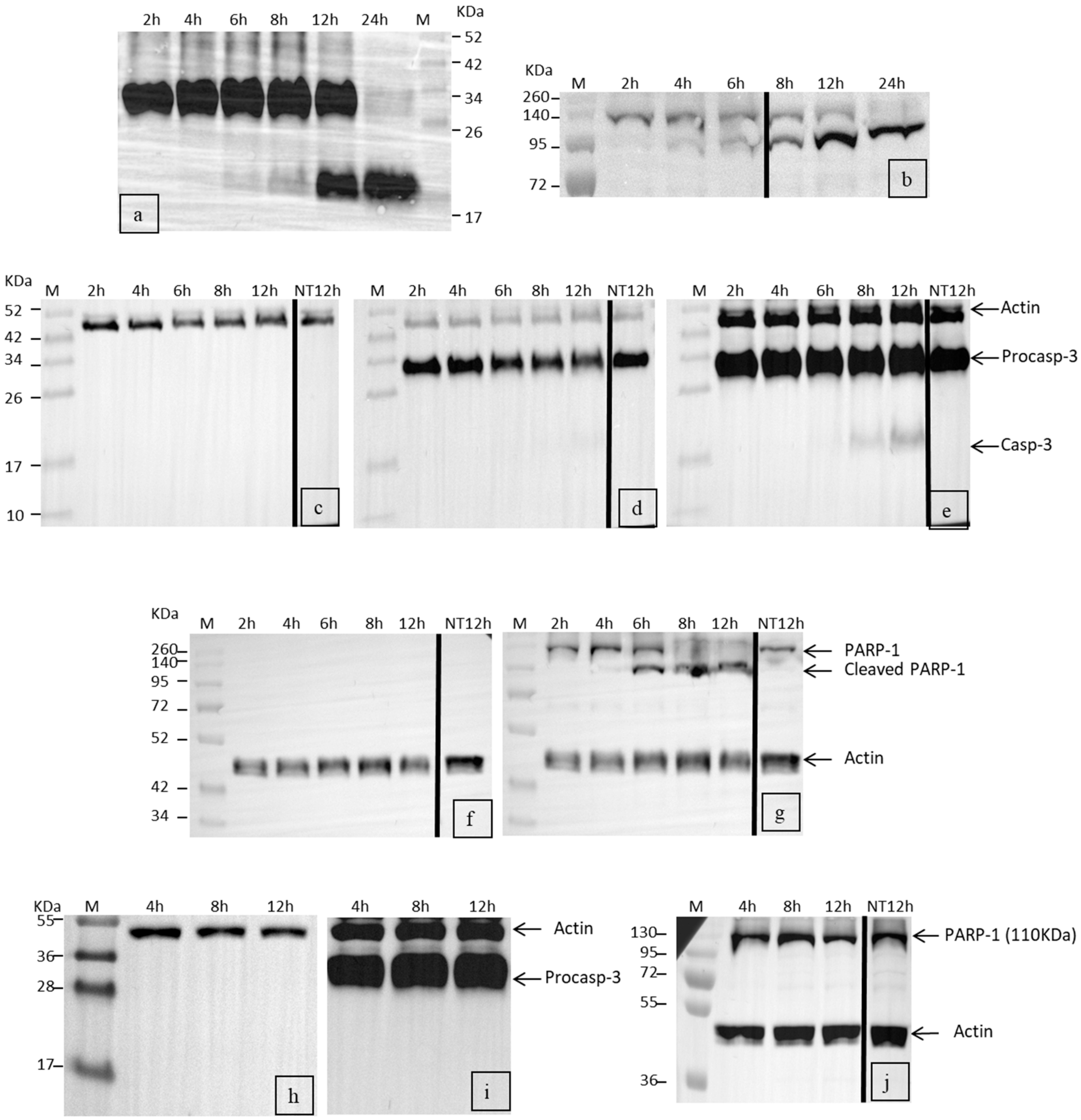

Figure S2. Full-length Western blot images given in Figure 3: positive control by treatment of 4T1 cells with staurosporine at 10µM showing Casp-3 activation (a) and PARP-1 cleavage (b); Effect of DSS aqueous extract on 4T1 cells (c, d, e, f, g): Successive labelling of 42KDa actin (c), 35KDa procaspase-3 and 17KDa cleaved caspase-3 (d,e) performed on blots obtained from 14% SDS-PAGE analysis of cell lysates, (f, g) detection of actin and PARP-1 antibody (116 and 89KDa) in cell lysates migrated on 10% SDS-PAGE before blotting. Evaluation of the effect of DSC aqueous extract on 4T1 cells (h, i, j) where (h, i) corresponds

to the same gel used to detect both actin and caspase-3; (j) Labelling of actin and PARP-1 on cell lysate analysed on 10% SDS-PAGE before blotting.

To detect the 17KDa fragment of activated caspase-3, blots were exposed for longer time (20 min).

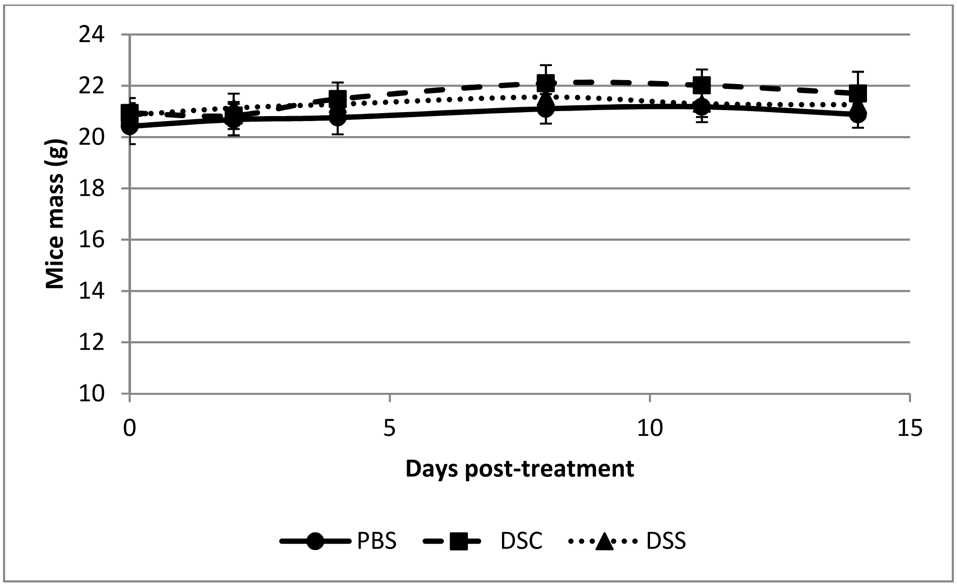

Figure S3. Weights of Balb/c bearing breast cancer following injection of 4T1 cells and treatment with DSC and DSS aqueous extracts at 5 mg/kg. Results shown are mean values ± Standard Error of the Mean.
